# A Wettability Contrast SERS Droplet Assay for Multiplexed Analyte Detection

**DOI:** 10.1021/acs.analchem.4c00831

**Published:** 2024-05-23

**Authors:** Vineeth Puravankara, Aravind Manjeri, Manish M. Kulkarni, Yasutaka Kitahama, Keisuke Goda, Prabhat K. Dwivedi, Sajan D. George

**Affiliations:** † Centre for Applied Nanosciences (CAN), Department of Atomic and Molecular Physics, Manipal Academy of Higher Education, Manipal 576104, India; ‡ Centre for Nanosciences, Indian Institute of Technology Kanpur, Kanpur 208016, India; § Department of Chemistry, The University of Tokyo, Tokyo 113-0033, Japan

## Abstract

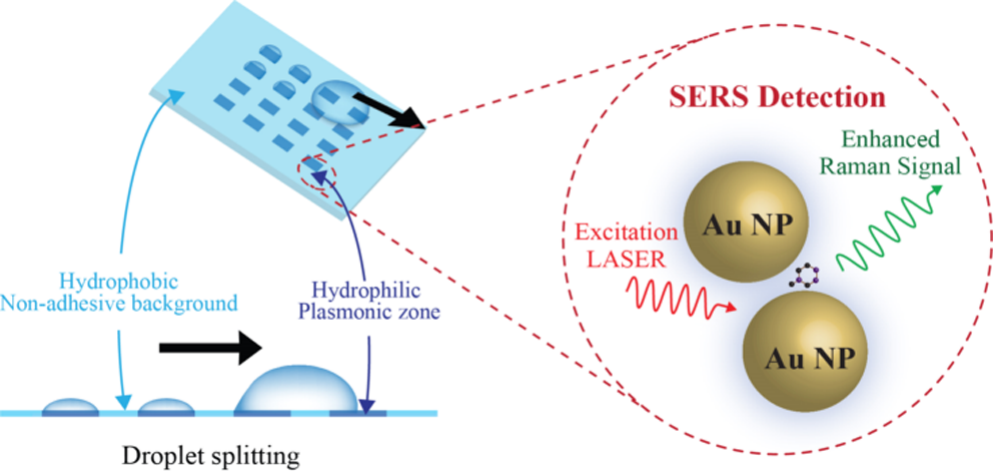

Droplet assay platforms have emerged as a significant methodology, providing distinct advantages such as sample compartmentalization, high throughput, and minimal analyte consumption. However, inherent complexities, especially in multiplexed detection, remain a challenge. We demonstrate a novel strategy to fabricate a plasmonic droplet assay platform (PDAP) for multiplexed analyte detection, enabling surface-enhanced Raman spectroscopy (SERS). PDAP efficiently splits a microliter droplet into submicroliter to nanoliter droplets under gravity-driven flow by wettability contrast between two distinct regions. The desired hydrophobicity and adhesive contrast between the silicone oil-grafted nonadhesive hydrophilic zone with gold nanoparticles is attained through (3-aminopropyl) triethoxysilane (APTES) functionalization of gold nanoparticles (AuNPs) using a scotch-tape mask. The wettability contrast surface facilitates the splitting of aqueous droplets with various surface tensions (ranging from 39.08 to 72 mN/m) into ultralow volumes of nanoliters. The developed PDAP was used for the multiplexed detection of Rhodamine 6G (Rh6G) and Crystal Violet (CV) dyes. The limit of detection for 120 nL droplet using PDAP was found to be 134 pM and 10.1 nM for Rh6G and CV, respectively. These results align with those from previously reported platforms, highlighting the comparable sensitivity of the developed PDAP. We have also demonstrated the competence of PDAP by testing adulterant spiked milk and obtained very good sensitivity. Thus, PDAP has the potential to be used for the multiplexed screening of food adulterants.

## Introduction

In recent years, the demand for rapid, sensitive, and cost-effective analytical methods has surged across various scientific and clinical disciplines. Responding to this growing need, droplet assay platforms have gained considerable attention for their ability to compartmentalize biological or chemical samples into submicron-volume droplets, allowing for individualized analysis with exceptional sensitivity.^1^ While beholding great promise, especially in lab-on-a-chip devices for point-of-care applications, practical applications of droplet assay platforms have been primarily confined to laboratory settings due to limitations in specialized instrumentation for consistent generation, control, and analysis of multiple droplets.^2^ Researchers traditionally rely on microfluidic platforms for droplet creation and manipulation in droplet assays. Achieving precise droplet splitting often requires sophisticated mechanisms, such as intricate valve designs, external techniques like surface acoustic waves,^3^ electrosplitting,^4^ and wettability contrast substrates prepared through advanced methods like laser patterning^5^ and photolithography.^6^ These methods demand complex instrumentation and are costly. Therefore, there is a need to develop a cost-effective and facile approach to facilitating droplet splitting into smaller volumes.

Wettability contrast droplet assay platforms, exploring differences between regions on a single substrate, offer a compelling alternative to high-instrumentation counterparts. These platforms utilize surface wettability to create precise microdroplets for diverse assay applications, making them low-cost and user-friendly. Recent advancements in microfluidic droplet manipulation platforms have demonstrated their ability to generate hundreds of droplets under various conditions, expanding the versatility and potential applications of wettability contrast techniques.^7^ Inspired by nature, researchers attempt to mimic the special wettability exhibited by different organisms.^8,9^ In a recent review, we summarized progress in the development of sensing platforms utilizing wettability contrast for droplet manipulation.^9^ Several platforms draw inspiration from superhydrophobic/superhydrophilic wettability contrast patterns found in the Stenocara desert beetle.^10^ Hydrophobic regions act as a barrier for droplets in the hydrophilic regions, preventing cross-contamination of samples. Moreover, the high adhesion force of superhydrophilic regions facilitates the partitioning of micrometer-sized droplets into submicron droplets. Hydrophobic/hydrophilic patterns can be fabricated with materials such as glass, quartz, polydimethylsiloxane (PDMS), and TiO_2_ via approaches such as UV exposure, plasma etching, and laser patterning.^11^ Despite the feasibility of splitting microliter-sized droplets, incorporating analytical platforms onto the substrate region below the daughter droplet is challenging. Our previous work reported the facile fabrication of a paper-based surface-enhanced Raman spectroscopy (SERS) droplet assay for analyte sensing.^12^ The platform utilizes the high difference in wettability between superhydrophobic candle soot-coated PDMS and superhydrophilic filter paper regions to enable the self-partitioning and confining of microdroplets. With the incorporation of plasmonic nanoparticles, SERS studies were conducted for methyl orange, achieving a limit of detection (LOD) down to a pM concentration. Many wettability contrast droplet assays have been integrated and adapted to serve as multiplexed SERS assays, representing a significant stride toward more comprehensive and efficient analytical solutions.

Among analytical platforms, SERS-based substrates have gained prominence in diverse fields due to their high sensitivity and specificity, making them powerful candidates for trace analyte detection.^13,14^ Recent advancements have witnessed the integration of these substrates into flexible materials such as PDMS, paper,^15^ and polyethylene, facilitating conformal contact with nonplanar and irregular surfaces, including human skin. This adaptability has paved the way for exploring SERS-based wearable sensors for real-time, noninvasive monitoring of diverse biochemical markers and components present in human sweat.^16^ Multiplexed analysis using SERS droplet assays offers advantages in the field of analytical chemistry, as it enables the simultaneous detection of multiple analytes within a single assay, dramatically increasing efficiency and throughput. Vedelago and his team recently developed a multiplex SERS micro assay capable of detecting specific variants of SARS-CoV-2.^17^ A similar work by Wu and co-workers developed a highly uniform SERS platform and demonstrated its rapid diagnosing capability for the detection of SARS-CoV-2.^18^ Another work developed a multiplexed assay using SERS that can perform rapid and accurate detection of bacterial resistance to β-lactam antibiotics. Recently, microfluidic SERS platforms have emerged as a prominent technique among SERS-based sensing strategies, owing to their exceptional sensitivity for detecting ultralow analyte concentrations in microscale environments. For instance, in a study by Zhang et al., a droplet-based microfluidic SERS system demonstrated rapid and effective detection of 6-thioguanine in serum, achieving significantly reduced detection times of around 10 s.^19^ Despite their advantages, current multiplexed SERS arrays have drawbacks, including complex fabrication procedures, the potential for signal interference between analytes, and sensitivity to environmental variations. This underscores the necessity of creating straightforward and reliable droplet assay platforms with simple fabrication procedures and robust stability, ensuring the ease of operation and capability of performing multiplexed SERS assay-based detection.

In this study, we present an innovative and facile approach to create nonadhesive wettability contrast plasmonic droplet assay platform (PDAP) SERS substrates to exploit multiplexed screening of food adulterants. The fabrication relies on the selective incorporation of plasmonic gold nanoparticles (AuNPs) onto a nonadhesive region of the hydrophobic quartz surface achieved via oil grafting. The resulting wettability contrast and adhesive properties between the oil-grafted and nanoparticle-coated regions enable the controlled splitting of a microliter-sized analyte droplet into smaller submicroliter droplets. Unlike the techniques discussed above, the fabrication of this substrate is significantly more straightforward and does not necessitate complex instrumentation. The droplet splitting process is purely gravity-driven, eliminating the need for external stimuli or force. We explore the potential of the developed substrate for the droplet splitting of aqueous solutions. To demonstrate the practical utility of PDAP for SERS studies, we conducted SERS analysis on dye molecules, such as Rhodamine 6G (Rh6G) and Crystal Violet (CV). Additionally, as a validation of its applicability as a droplet assay technique for practical application, we applied this platform to the identification of multiple adulterants in milk samples using SERS-based methods.

## Experimental Section

### Chemicals and Materials

Quartz slides were purchased from Swetech, India. The silicone oil (100 cst), gold chloride(III) trihydrate ≥99% (HAuCl_4_·3H_2_O), and rhodamine 6G (95%) were purchased from Sigma-Aldrich Co., Ltd. Trisodium citrate dihydrate (≥99%), urea, ammonium sulfate, etc. were purchased from Merck Life Science Pvt. Ltd. Melamine (extra pure) and crystal violet (88%) were purchased from Loba Chemie Pvt. Ltd. Milk was purchased from local supermarkets.

### Synthesis of Gold Nanoparticles (AuNPs)

All of the glassware and quartz slides were cleaned using aqua regia solution (a mixture of hydrochloric acid and nitric acid at a volume ratio of 3:1), followed by rinsing with copious amounts of deionized (DI) water, and were dried in air at ambient conditions. AuNPs were synthesized using the previously reported citrate reduction method.^16^ 50 mL of 1 mM HAuCl_4_·3H_2_O was heated at 90 °C. To this, 40 mM of trisodium citrate solution was added quickly. The solution was magnetically stirred continuously until the color of the solution turned from black to wine red. The obtained nanoparticle solution was then cooled to room temperature and stored at 4 °C.

### Wettability Contrast Plasmonic Droplet Assay Platform (PDAP) Fabrication

**Figure 1:**
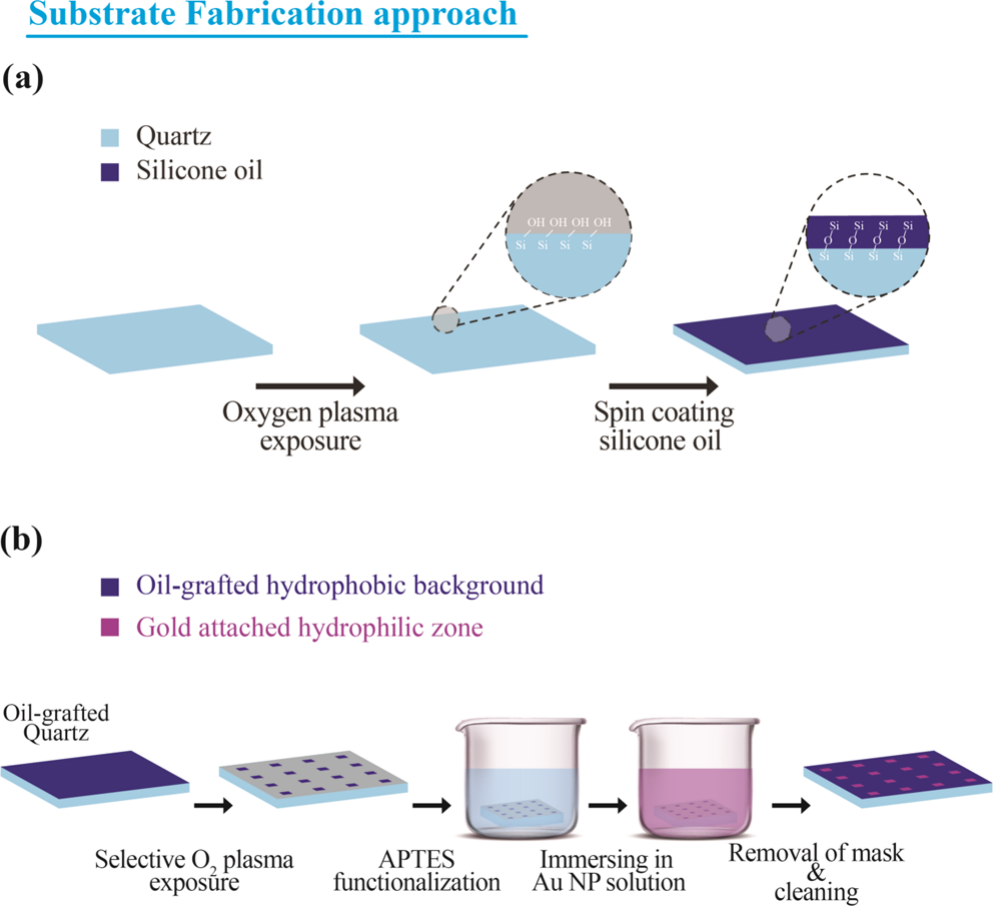
(a) Schematic for fabricating the hydrophobic nonadhesive quartz surface. (b) Selective functionalization with plasmonic gold nanoparticles for creating the droplet assay platform.

A nonadhesive quartz surface was fabricated by grafting silicone oil onto the quartz substrate with the aid of oxygen plasma reported in our previous works.^20,21^ Initially, the quartz slide was cleaned using piranha solution (H_2_SO_4_ and H_2_O_2_, 3:1), rinsed with water, and then dried in ambient air. The silicone oil grafting onto the cleaned substrate was carried out by exposing the substrate to oxygen plasma from a commercial oxygen plasma (Femto low-pressure plasma system, Diener electronic) at 90 W for 60 s at a 0.25 mbar pressure. Following the oxygen plasma exposure to the quartz substrate, 1 mL of 100 cst silicon oil is spin-coated onto the substrate and kept overnight to initiate the grafting of oil onto the quartz surface. The excess oil was removed by the ultrasonication process using Milli-Q water and soap solution. Figure 1a depicts a schematic of the oil grafting method.

For creating a plasmonic droplet assay platform for SERS studies, plasmonic nanoparticles were selectively incorporated into the prepared nonadhesive quartz substrate. For this, a method of functionalizing plasmonic AuNPs onto the quartz was employed via (3-aminopropyl) triethoxysilane (APTES) functionalization. First, onto a silicone oil-grafted quartz substrate, masks made out of scotch tape with exposed rectangular zones of dimension about 1 × 1 mm^2^ were placed. The unmasked regions of the substrate were silanized with APTES by exposing the substrate to oxygen plasma with a power of 90 W for 60 s, followed by dipping in 10% APTES solution in ethanol for 2 h. The silanized quartz was then rinsed with excess ethanol before they were dried in an oven at 80 °C for 1 h. The silanized glass slides were then dipped into 20 mL of synthesized AuNP solution for 24 h for the particles to get attached to the silanized surface. After 24 h, the substrate was taken out from the solution rinsed with water and dried. The masks were carefully removed from the substrate. Figure 1b shows a schematic of the fabrication procedure.

### Raman Instrumentation

The Raman spectroscopic studies were carried out using a lab-built fiber-based Raman setup. In brief, a 785 nm diode laser (Ondax) is used as the excitation source. A laser beam with a maximum output power of 175 mW was passed through a variable neutral density filter (Holmarc, India) to adjust the laser power levels. An optical isolator was inserted in the laser beam path to avoid the backscattered light to the laser source. The beam was then focused into the excitation fiber of diameter 105 μm of a Raman fiber probe (BAC 102, B&WTek) using a biconvex lens of 5 cm ensuring the f matching. The sample of interest was placed at the other end of the fiber probe. The Raman scattered signals were collected by the collection fiber of the probe having 200 μm diameter and are directed to a charge coupled device (CCD)-coupled spectrograph (Kymera 193i, Andor). The Raman probe is equipped with a low-pass edge filter that eliminates the high-intensity Rayleigh signals. Supporting Figure S1 shows the schematic of the lab-built fiber-based Raman setup. The analyte droplets were split across the hydrophilic regions (where the plasmonic particles are attached) of the wettability contrast plasmonic droplet assay substrate. The Raman spectral measurements were carried out from five different hydrophilic regions to confirm the reproducibility of the measurements and to calculate the relative standard deviation (RSD) values. The SERS signals from Rh6G and CV were collected with a laser power of 30 mW and exposure times of 30 and 20 s, respectively.

### Measurement Techniques

The UV–vis absorption spectra of the prepared AuNP solution were acquired using a 190–900 nm UV–vis spectrometer (JASCO V-650). The average particle size and ζ potential were measured using a nanoparticle analyzer (Horiba SZ-100). Field emission scanning electron microscopy (FESEM, ZEISS, Supra 40 VP) was carried out to investigate the surface morphology and confirm the presence of plasmonic nanoparticles on the surface. To confirm the particle dimension and shape of the nanoparticle, high-resolution transmission electron microscopy (HRTEM) was carried out using FEI-Titan, G-2, 60–300 kV HRTEM, equipped with a high-angle annular dark-field (HAADF) detector for elemental mapping. The water contact angle (WCA) measurements are carried out using a commercial contact angle instrument (Holmarc, India).

### Milk Adulterant Samples Preparation for SERS Testing

In this study, we used locally purchased cow milk as our primary test matrix. To investigate concentration-dependent effects, we prepared aqueous solutions of three frequently encountered milk adulterants: urea, ammonium sulfate, and melamine. This was achieved by carefully adding the powdered adulterants into water in precise quantities, resulting in solution concentrations of 10, 8, 6, 4, and 2 mg/mL. Adulterated milk solutions were prepared by dissolving the appropriate amount of powdered adulterant in milk. Similar to the previous case, the concentration of adulterants varied from 2 to 10 mg/mL. Another set of adulterated milk solutions was prepared by mixing all three adulterants with 1 mL of a milk solution in an equal ratio.

For the Raman spectroscopic analysis of these samples, a 10 μL droplet of each sample was split across the hydrophilic regions of the contrast substrate, achieved by placing the substrate at an inclination angle exceeding 30°. Raman spectra were acquired over the spectral range of 400–1700 cm^–1^, encompassing the characteristic Raman peaks of both milk and potential adulterants. Consistent experimental conditions were maintained with a laser power of 30 mW and an exposure time of 30 s for all milk and adulterant samples.

## Results and Discussion

### Characterization of AuNPs

**Figure 2:**
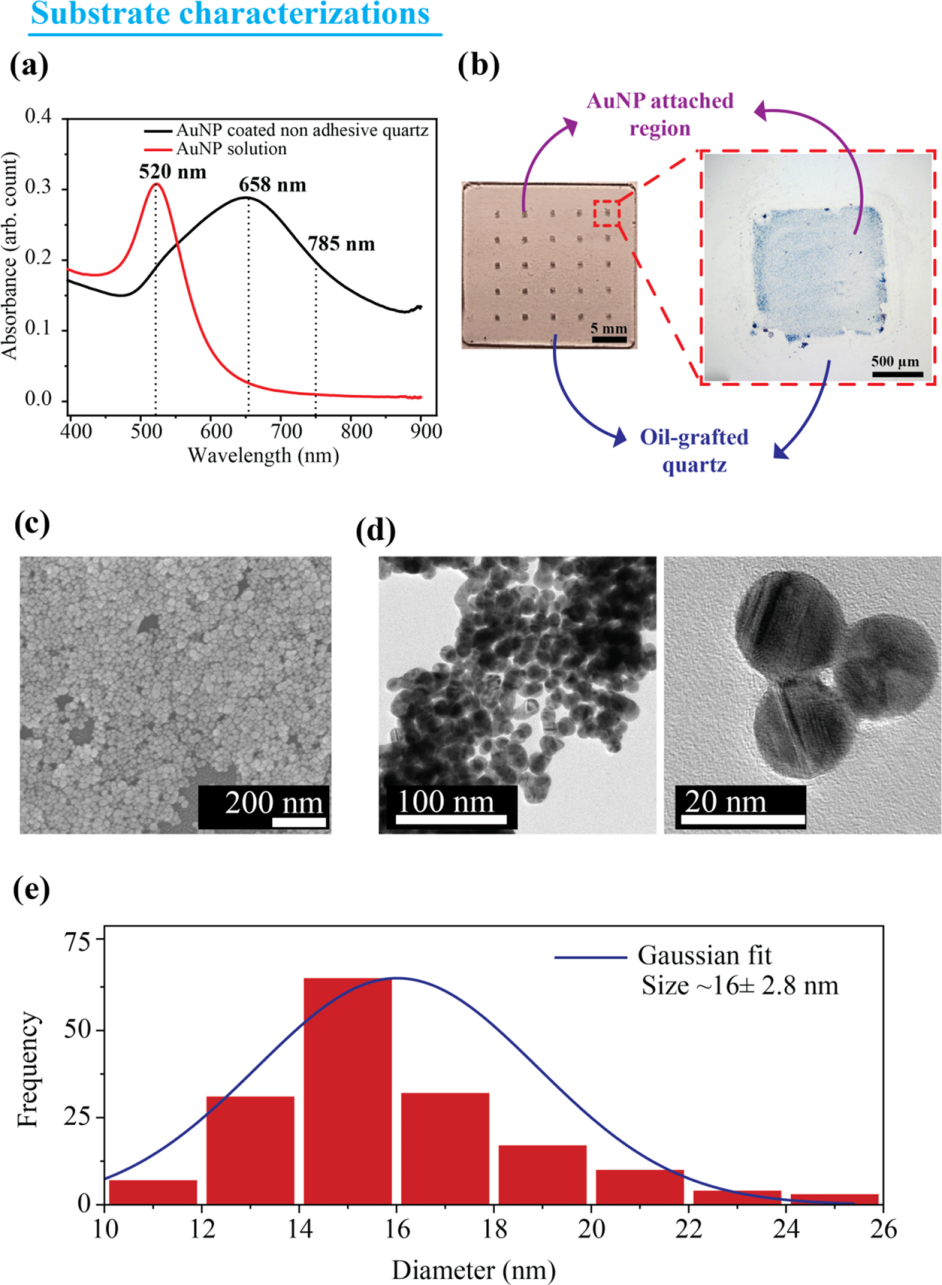
(a) UV–visible absorption spectra of AuNP solution as well as AuNP-coated nonadhesive quartz substrate. (b) A photograph of the fabricated plasmonic droplet assay substrate and the enlarged microscopic images showing the zones decorated with AuNPs and the oil-grafted quartz region on the substrate. (c) FESEM images of the AuNP-attached region on the substrate, (d) the HRTEM image of the prepared nanoparticles with different magnifications, and (e) the corresponding particle size distribution fitted to a Gaussian distribution.

The average particle size and ζ potential of the synthesized Au nanoparticles are characterized using the Horiba particle size measurement system and are measured to be ∼52.4 nm and −30.7 mV, respectively. The UV–vis absorption spectrum shown in Figure 2a illustrates the absorption spectrum of gold colloids with a plasmonic peak of ∼520 nm. However, the incorporation of the plasmonic particles onto the transparent nonadhesive quartz broadens the plasmonic spectra and peaks to 658 nm due to the coupling of the plasmonic field of adjacent nanoparticles. The efficient selective incorporation of plasmonic nanoparticles via APTES functionalization through the scotch tape mask results in a color change as shown in the optical image (Figure 2b) upon removal of the scotch tape. The enlarged microscopic image of the single cell region of the fabricated substrate shown in Figure 2b reveals that there is a fairly good contrast between the AuNP-attached regions and the oil-grafted regions. An FESEM image analysis of the nanoparticle-incorporated region (Figure 2c) illustrates the close packing of plasmonic nanoparticles. An approximate size of the particle is calculated from the FESEM image as 20.7 ± 1.7 nm. The HRTEM image of the prepared nanoparticle given in Figure 2d confirms that the particles possess a spherical shape. The frequency distribution of sizes was found to be unimodal and was fitted to a Gaussian with an average of ∼16 nm and a standard deviation of 2.8 nm (Figure 2e). This result is in good agreement with the FESEM results. In addition, the HAADF images of elemental mapping shown in Supporting Figure S2 confirm that the signal arises from AuNPs.

### Nonadhesive Wettability Contrast for Droplet Splitting

**Figure 3:**
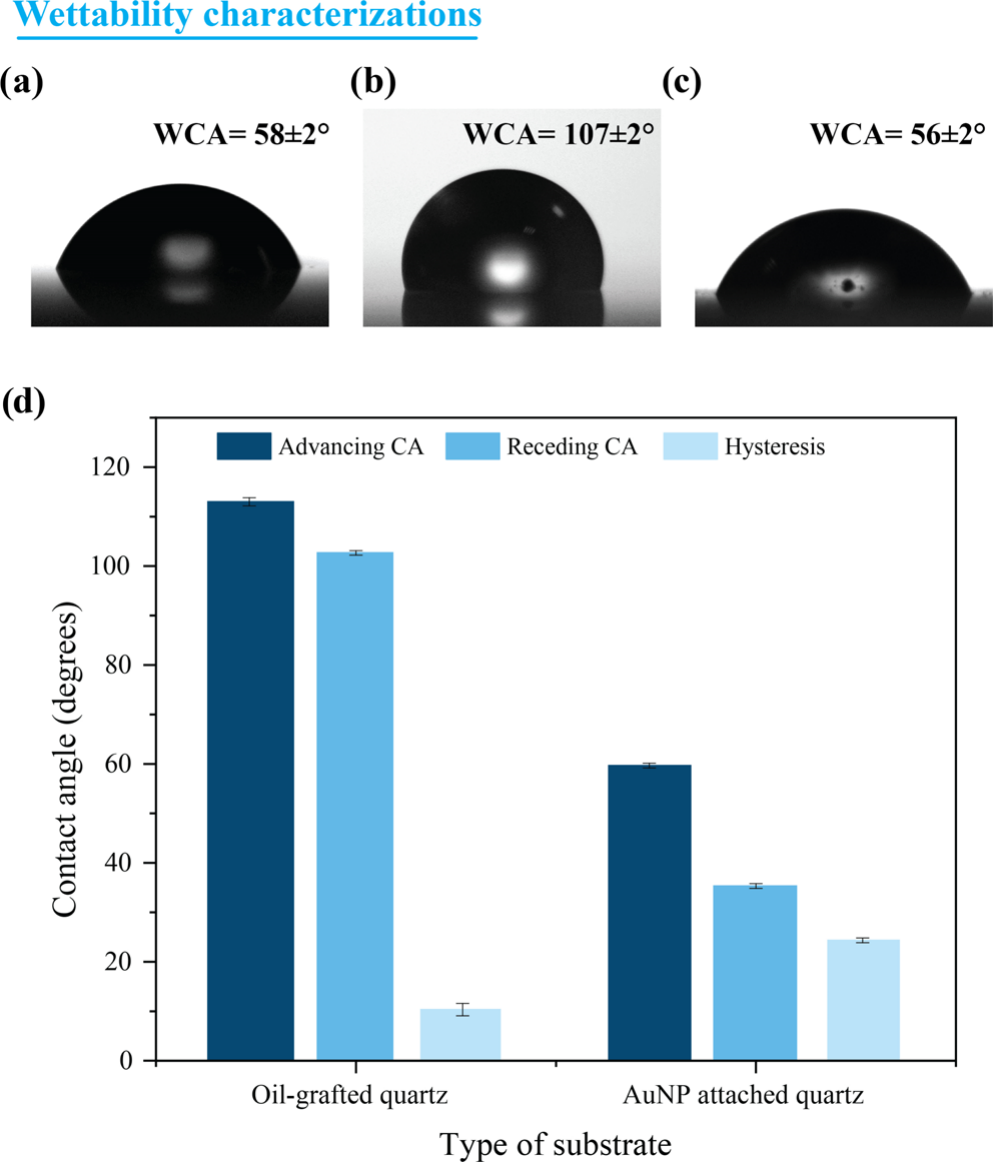
Contact angles of (a) pristine quartz, (b) oil-grafted quartz, and (c) AuNP-attached quartz substrates. (d) The advancing CA, receding CA, and CA hysteresis of these substrates.

A nonadhesive coating on a quartz substrate is achieved via silicone oil grafting method as depicted in Figure 1a. The exposure of oxygen plasma onto the cleaned quartz surface results in the formation of −OH groups on the surface and makes them highly hydrophilic (with a water contact angle, WCA ∼ 0°). The −OH bonds on the quartz surface facilitate the grafting of silicone oil onto the surface via hydrolysis and condensation reactions.^22,23^ As a result of oil grafting, the hydrophilic quartz becomes hydrophobic with a nonadhesive behavior toward the water. The equilibrium water contact angle (WCA) of a 10 μL droplet on the oil-grafted surface is measured to be ∼107 ± 2°, as compared with the pristine quartz that exhibits a WCA of 58 ± 2° (Figure 3a,b). The oil grafting converted the intrinsically hydrophilic quartz not only to hydrophobic but also nonadhesive toward the water. The nonadhesive nature of the silicone oil-grafted surface to a water droplet is illustrated by the spontaneous movement of a 10 μL droplet dispensed onto the substrate upon a tilt angle of 30° (Supporting Video SV1). On the other hand, a 10 μL water droplet adhered to the pristine quartz substrate even for a tilt angle of 180°. To ensure the uniformity of the grafted oil film, the WCA values were measured at 10 different positions on the oil-grafted surface and the measured values were found to be nearly the same.

The wettability contrast surface fabricated onto the nonadhesive silicone oil-grafted surface achieved by following the APTES-mediated functionalization depicted in Figure 1b results in the formation of AuNPs. Here, the self-organization of AuNPs is achieved through an electrostatic interaction between AuNPs and the APTES-functionalized quartz surface. It is a well-known linking agent of APTES consisting of three hydrolyzable ethoxy groups and an amine (NH_2_) that point away from the substrate. The oxygen plasma exposure enriches the surface with OH bonds and upon immersion in APTES solution, the Si bond from APTES results in the covalent bonding with the oxygen in the −OH-terminated surface oil-grafted surface, leaving the other end of the APTES molecule with a positively charged amine group.^24^ The positively charged amine group binds to the negatively charged gold nanoparticles via electrostatic interaction to create a layer of plasmonic AuNPs on the surface. Herein, the selective attachment of plasmonic AuNPs is achieved using a mask made of Scotch brand tape, which consisted of rectangular open regions of dimensions 1 × 1 mm^2^ cut out from it, as shown in Supporting Figure S3. This enables AuNPs to go and attach only to exposed locations of the silicone oil-grafted quartz substrate. Following the removal of the scotch tape after the AuNP attachment, the masked region exhibits its inherent nonadhesive nature toward water droplets due to the grafted oil whereas the unmasked region illustrates a hydrophilic nature due to the presence of gold nanoparticles. In comparison to the oil-grafted region (107 ± 2°), the water contact angle of the AuNP-attached region exhibits a reduced value of 56 ± 2° (Figure 3c).

Further, the difference in the adhesive nature of the nonadhesive oil-grafted region and the AuNP-attached region is estimated using the expression *F*_adh_ ≈ *w*γ(cos θ_r_ – cos θ_a_) where γ is the surface tension of the liquid, *w* is the base width of the droplet, and θ_r_ and θ_a_ are the receding and advancing contact angles, respectively. In our calculations, we have used the surface tension value for water, which is 72 mN/m. For a pristine glass substrate with a contact angle hysteresis of approximately 29° and a base width of 4.1 mm, the estimated *F*_adh_ is approximately 101 μN. On the other hand, the oil-grafted quartz surface, with a contact angle hysteresis of about 10° and a base width of 3.5 mm, exhibits a lower *F*_adh_ of approximately 42 μN. In the case of the AuNP-attached substrate, where the contact angle hysteresis is measured at around 25° and the base width is 4.6 mm, the estimated *F*_adh_ for the AuNP-attached substrate is approximately 106 μN, more than twice the adhesion force observed for the oil-grafted quartz substrate (Figure 3d).

**Figure 4:**
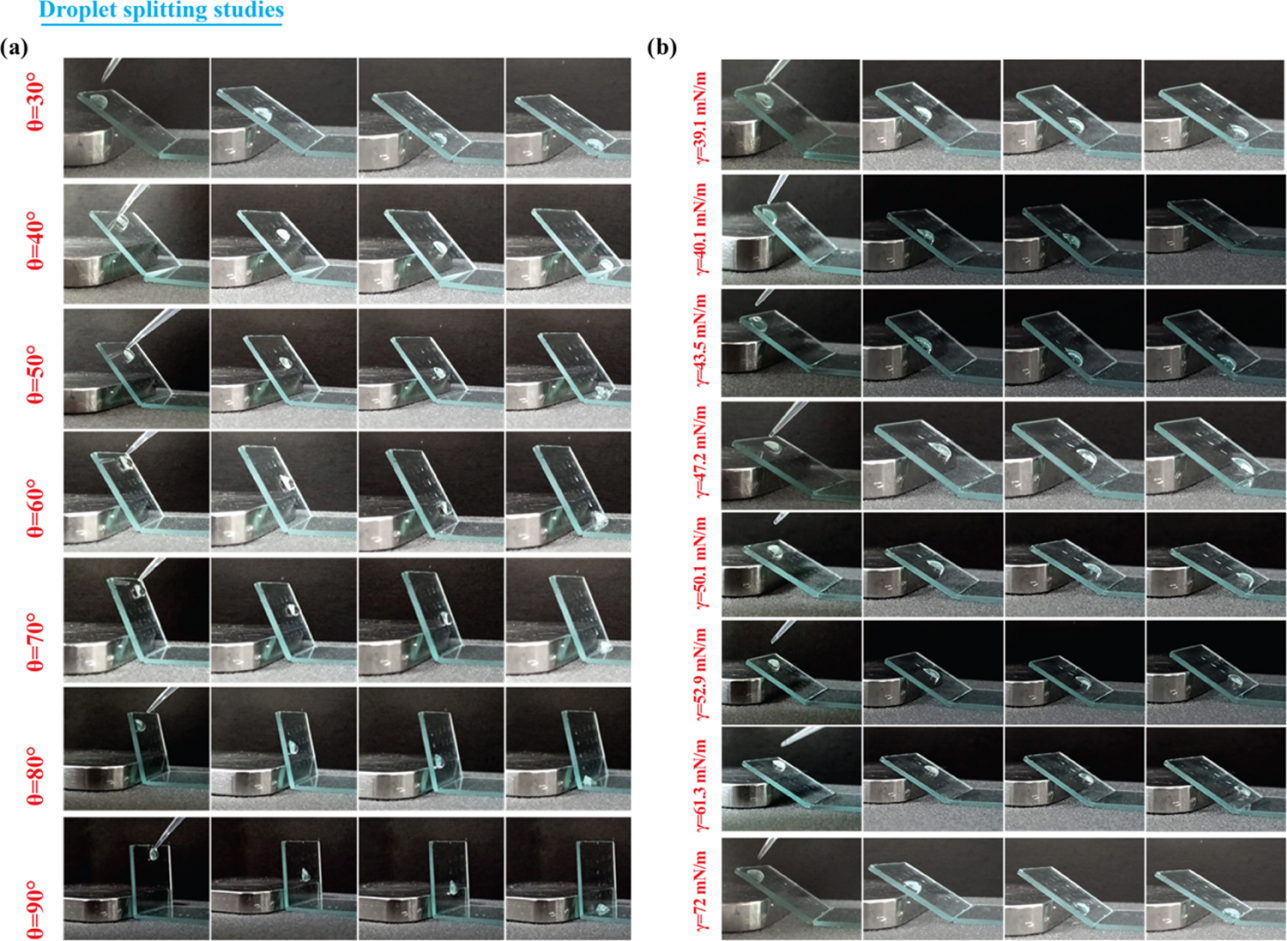
(a) Splitting of a 20 μL droplet on the fabricated wettability contrast substrate placed at different inclination angles ranging from 30 to 90°. (b) Droplet splitting of 20 μL solutions of different surface tension values.

Driven by the wettability contrast and difference in the force of adhesion between the oil-grafted regions and the AuNP-attached regions, a drop of water moving over the plasmonic droplet assay substrate was found to split into a smaller droplet and reside on the plasmonic region of the whole substrate. It has been observed that the master droplet split into daughter droplets for an inclination angle above 30°, as shown in Figure 4a (Supporting Video SV2). The volume of the daughter droplet is determined by the dimension of the hydrophilic zone. It is pertinent to mention here that the splitting of the droplet occurred under the gravity-driven flow of the master droplet and no additional external force is employed. The high contact angle hysteresis in the plasmonic region offers a higher pinning effect while the droplet moved across the surface compared with the oil-grafted region and thus led to the formation of the daughter droplet from the moving droplet. Though the hydrophilic zones are square in nature, using the spherical cap approximation formula,  where *a* and *h* are the base radium and height of the daughter droplet, respectively, and the volume of the daughter droplet is estimated for a 10 μL droplet of glycerol split across the plasmonic droplet assay. It has been observed that with a 10 μL droplet, it is possible to create daughter droplets of volume ∼0.12 ± 0.03 μL when the area of the hydrophilic zone dimension is 1 mm^2^.

Further, the role of surface tension of the liquid in the splitting process is investigated here. In this case, the tilt angle is kept at a fixed value of 300 and the measurements are carried out using a 20 μL droplet of solutions having surface tension ranging from 72 to 39.08 mN/m. As shown in Figure 4b and Supporting Video SV3, the plasmonic droplet assay platform is efficient in splitting the droplet over this wide range of surface tension. Herein, the surface tension of the liquid is varied by adding the surfactant cetyltrimethylammonium bromide (CTAB) of different concentrations to water. In addition to aqueous analyte solutions, the droplet splitting of complex body fluids such as saliva also was carried out, and it is observed that the saliva was split into smaller daughter droplets for inclination angles above 70° purely under the influence of gravity. The droplet splitting with saliva on the developed PDAP is shown in Supporting Figure S4. This demonstration proves the potential usability of our developed PDAP for body-fluid-analysis-based diagnostics, ensuring minimal usage of body fluids for study.

### SERS Performance of Rh6G and CV on PDAP

**Figure 5:**
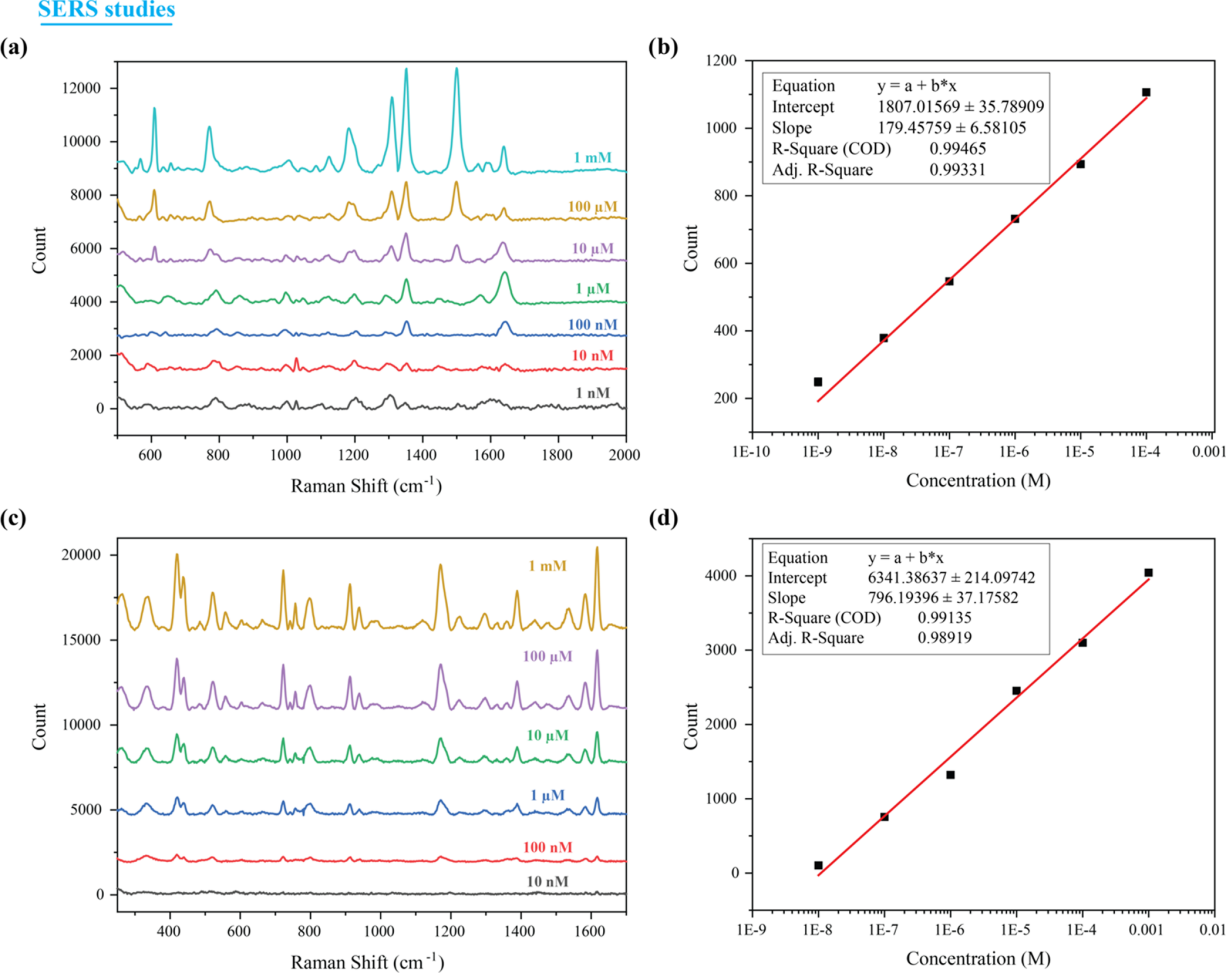
(a) Raman spectra of different concentrations of Rh6G and (b) linear variation in the signal intensity of the 1360 cm^–1^ Raman peak as a function of different concentrations of Rh6G. (c) Raman spectra of different concentrations of crystal violet and (d) linear variation in the signal intensity of the 1358 cm^–1^ peak as a function of different concentrations of CV.

The potential of the developed plasmonic substrate to fingerprint the chemical identity of the molecules via the SERS technique was investigated here by recording Raman spectra of the dye molecules Rhodamine 6G (Rh6G) and Crystal Violet (CV). For this, Rh6G and CV were of different concentrations such as 1 mM, 10 μM, 1 μM, 100 nM, 10 nM, and 1 nM, which were split across the plasmonic hydrophilic regions in contrast by keeping the substrate at angles above 30°. The Raman spectra of Rh6G and CV of varied concentrations were recorded, and the results are shown in Figure 5a. The obtained Raman spectral peaks corresponding to Rh6G agreed well with the literature, as given in Supporting Table ST1.^25,26^ As observed in Figure 3a, Rh6G exhibited distinct Raman spectral peaks up to a concentration of 1 nM onto the developed substrate. The limit of detection (LOD) for Rh6G was calculated by plotting the variation of intensity of the 1360 cm^–1^ Raman peak as a function of concentration (Figure 5b). Using the expression *y* = *Y*_B_ + 3*S*_B_, where *y* is the intensity count at zero analyte concentration, *Y*_B_ is the background signal, and *S*_B_ is the standard deviation corresponding to the background, LOD was estimated to be 134 pM. The relative standard deviation of the measurements is ∼13%.

Similar to Rh6G, the obtained Raman spectral peaks of CV also agreed with the literature,^26,27^ as shown in Supporting Table ST2. Herein, the CV Raman spectra showed distinct peaks up to a concentration of 10 nM (Figure 5c). LOD for CV was estimated by plotting the variation of intensity of the 1615 cm^–1^ Raman peak as a function of concentration (Figure 5d) and was calculated to be 10.1 nM. The relative standard deviation of the measurements was found to be ∼14%. To evaluate the Raman signal enhancement resulting from the plasmonic Au nanoparticles in the developed plasmonic substrate, the Raman spectra of Rh6G and CV were recorded on oil-grafted plain quartz as well as the AuNP-functionalized droplet assay substrates and compared. The obtained results shown in Supporting Figure S5 unambiguously demonstrated a significant enhancement in the Raman spectral signal counts for identical spectral excitation and recording parameters that account for about 9 and 10 times the signal magnitude for Rh6G and CV, respectively. The enhancement factor of the fabricated plasmonic substrate was determined by comparing the Raman signals of the dye concentrations on an oil-grafted quartz surface devoid of plasmonic nanoparticles. It was observed that the method successfully yielded enhancement factors of 10^7^ and 10^6^ for Rh6G and CV for a daughter droplet of 120 nL, respectively. A comparison of the detection levels of Rh6G and CV of various SERS substrates in some of the reported literature has been presented in Supporting Table ST3. This indicates that the SERS performance of the developed plasmonic droplet assay sensor is comparable to the reported values achieved through different fabrication approaches.

### Performance Evaluation of PDAP with Milk Adulterants

To further demonstrate the potential of the fabricated plasmonic droplet assay for multiplexed analyte detection, the detection of milk adulterants was demonstrated. Initially, the Raman spectra of commonly employed milk adulterants such as urea, ammonium sulfate (AmS), and melamine (Mel) were recorded, and the results are shown in Supporting Figure S6. It was observed that the Raman spectrum of the investigated samples agreed well with the literature, as shown in Supporting Tables ST4–ST6. It was observed that the mentioned adulterants have distinct Raman peaks to fingerprint the molecule. The strongest band of urea appeared at 1005 cm^–1^ and stems from the symmetric N–C–N stretching.^27,28^ The weaker peaks situated at 548, 1173, 1538, and 1648 cm^–1^ originated from vibrational modes such as CO deformation vibration, NH_2_ symmetric rocking, CO symmetric stretching, and NH_2_ symmetric deformation vibration, respectively.^29,30^ AmS exhibited a strong Raman peak at 976 cm^–1^, which corresponds to the symmetric stretch mode of SO_4_^2–^, while the other weaker peak was observed at 620 cm^–1^ due to the triply degenerate SO_4_^2–^ deformation mode. In the case of melamine, the strongest peak was observed at 679 cm^–1^, which stems from the in-plane deformation of the triazine ring.^31^ The additional weaker Raman peak at 986 cm^–1^ can be attributed to the ring-breathing mode resulting from the C–N stretching of the triazine ring.

To evaluate the applicability of the substrate for Raman studies of milk adulterants, aqueous solutions of different concentrations of the adulterants (10, 8, 6, 4, and 2 mg/mL) were prepared and partitioned across the plasmonic hydrophilic regions of the wettability contrast substrate. All of the Raman spectra were recorded with a laser excitation power of 30 mW and an exposure time of 30 s. The Raman spectra observed for urea with various concentrations are shown in Supporting Figure S7 and it is evident from the figure that as the concentration varied from 2 to 10 mg/mL, the peak at 1005 cm^–1^ exhibited a pronounced variation in intensity. Additionally, the peak at 548 cm^–1^ also displayed a similar trend. A similar variation was observed in the Raman spectral intensity of 620 as well as 976 cm^–1^ peaks of ammonium sulfate as the analyte concentration changed (Supporting Figure S8). The Raman peaks of melamine situated at 376, 679, and 986 cm^–1^ also exhibited a similar variation in the signal intensity depending on the varying concentration (Supporting Figure S9) and thus implies that the characteristic peak of the adulterant varies with the concentration.

LOD of individual milk adulterants on the developed PDAP was estimated in a similar way discussed in the previous section by plotting the variation of intensity of prominent Raman peaks of urea (1005 cm^–1^), ammonium sulfate (976 cm^–1^), and melamine (679 cm^–1^) as a function of concentration (Supporting Figures S7–S9). The limit of detection for milk adulterant solutions on the developed substrate is estimated to be 0.211 mg/mL for urea, 0.226 mg/mL for melamine, and 0.252 mg/mL for ammonium sulfate. The developed PDAP could detect urea at a level below the maximum permissible limit of 0.7 mg/mL.^32^ Though there is no set standard reported for ammonium sulfate in milk, the maximum permissible limit of sulfate ion in drinking water is 0.4 mg/mL and the developed substrate could achieve an appreciable limit of detection below the maximum permissible limit.^33,34^ On the other hand, although the allowable limit of melamine in milk is exceptionally low, up to 0.0025 mg/mL,^35^ the developed PDAP was able to achieve a commendable LOD of up to 0.226 mg/mL through a facile and label-free approach.

**Figure 6:**
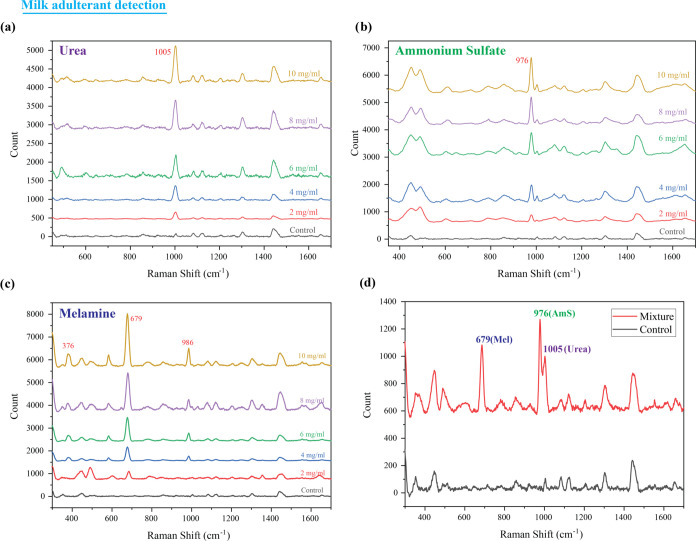
Raman spectra of milk spiked with different concentrations of milk adulterants: (a) urea, (b) ammonium sulfate, and (c) melamine. (d) Comparison of Raman spectra of pure milk with the milk mixed with all three adulterants.

The milk samples were subjected to adulteration through the addition of various quantities of powdered milk adulterants, including urea, ammonium sulfate, and melamine, at concentrations ranging from 2 to 10 mg/mL. Figure 6a illustrates the Raman spectra of milk samples spiked with varying concentrations of urea (10, 8, 6, 4, and 2 mg/mL). As evident from Figure 6a, the Raman intensity of the prominent peak of urea, located at 1005 cm^–1^, corresponding to the symmetric N–C–N stretching, exhibited an adulterant urea concentration-dependent variation in the Raman signal. These results were in concurrence with the studies carried out in aqueous medium. Similarly, in the case of ammonium sulfate, the major peak of melamine at 976 cm^–1^ showed a variation in its peak intensity (Figure 6b). Three peaks of melamine situated at 376, 679, and 986 cm^–1^ showed a concentration-dependent variation of the Raman signal intensity (Figure 6c). The milk sample was subsequently blended with an equal ratio of urea, ammonium sulfate, and melamine. For the preparation of this mixture, we standardized the concentration of each adulterant to 6 mg/mL, representing a midrange value within the concentration range explored in this study. Remarkably, the Raman spectra of the milk mixture revealed distinct peaks corresponding to all three adulterants: 1005 cm^–1^ for urea, 976 cm^–1^ for ammonium sulfate, and 679 cm^–1^ for melamine as shown in Figure 6d. Notably, these peaks were absent from the control spectrum. It is worth emphasizing that our developed SERS substrate demonstrates exceptional sensitivity in detecting these adulterants at concentrations as low as 2 mg/mL, which is commonly encountered in adulterated milk within the market. Thus, the developed system provides an opportunity for sensitive and multiplexed analyte detection of a low concentration of sample volume.

## Conclusions

In summary, our work presents the development of a novel nonadhesive plasmonic droplet assay platform (PDAP) that leverages wettability contrast between two distinct regions to achieve droplet splitting. Initially, we modify a nonadhesive quartz substrate through oil grafting, enhancing its functionality by selectively integrating plasmonic gold nanoparticles. The contrast in wettability characteristics and adhesion properties between the nanoparticle-attached and oil-grafted areas facilitates the spontaneous division of a microliter-sized droplet into submicroliter droplets as it traverses different regions on the substrate. Remarkably, this splitting approach operates solely under the influence of gravity, mitigating the need for any external agents. Through the integration of plasmonic gold nanoparticles, the wettability contrast substrate transforms into a promising surface-enhanced Raman spectroscopy (SERS) platform, suitable for precise molecular fingerprinting of various analytes. The effectiveness of this substrate in SERS investigations was convincingly demonstrated using rhodamine 6G and crystal violet, resulting in impressive limits of detection (LOD) of 134 pM and 10.1 nM, with enhancement factors of ∼10^7^ and ∼10^6^, respectively. Furthermore, in an application aimed at droplet assays, the substrate proved its utility in the detection of milk adulterants at different concentrations using the SERS technique. Based on the outcomes of this study, we envision that the innovative Raman-spectroscopy-integrated plasmonic droplet assay platform developed here holds significant potential for a wide range of multiplexed analytical applications. These applications could span high-throughput drug screening, cancer research, diagnostic kits for diseases using body fluids, biotechnology, and beyond.

## Supporting Information Available

Schematic of the lab-built-based Raman setup (S1), HRTEM-HAADF elemental analysis of the prepared nanoparticles (S2), photograph of the Scotch-tape mask used for selective attachment of AuNPs (S3), droplet splitting of the saliva on PDAP at different inclination angles (S4), comparison of Raman spectra of dyes on plain quartz as well as developed plasmonic droplet assay platform (S5), Raman spectra of milk adulterants in pure form (S6), Raman spectra of different concentrations of urea (S7), Raman spectra of different concentrations of ammonium sulfate (S8), Raman spectra of different concentrations of melamine (S9), Raman peak assignments of rhodamine 6G (Table ST1), Raman peak assignments of crystal violet (Table ST2), comparison of detection levels of Rh6G and CV (Table ST3), Raman peak assignments of urea (Table ST3), Raman peak assignments of ammonium sulfate (Table ST4), and Raman peak assignments of melamine (Table ST5) (PDF)
Movement of a 10 μL droplet on the oil-grafted quartz substrate at an inclination of 30° (MP4)
Droplet splitting on PDAP at different inclination angles (MP4)
Droplet splitting on PDAP with different surface tension liquids (MP4)
