# Supplementary material for: A Wettability Contrast SERS Droplet Assay for Multiplexed Analyte Detection
Source: Anal Chem. 2024 May 23;96(22):9141–50. doi: 10.1021/acs.analchem.4c00831 (PMC11154665; doi:10.1021/acs.analchem.4c00831)
Supplement: Supplementary file 1 — ac4c00831_si_001.pdf [file ac4c00831_si_001.pdf]

Supporting Information

A wettability contrast SERS droplet assay for multiplexed analyte detection

Vineeth Puravankara[1], Aravind Manjeri[1], Manish M. Kulkarni[2], Yasutaka Kitahama[3], Keisuke Goda[3], Prabhat K. Dwivedi*[2], Sajan D. George*[1]

[1]Centre for Applied Nanosciences (CAN), Department of Atomic and Molecular Physics, Manipal Academy of Higher Education, Manipal, India – 576104

[2]Center for Nanosciences, Indian Institute of Technology Kanpur, Kanpur (UP) 208016, India.

[3]Department of Chemistry, The University of Tokyo, Tokyo, 113-0033 Japan

Corresponding authors' emails: *sajan.george@manipal.edu, *prabhatd@iitk.ac.in

Table of contents

S.No.	Topic	Page No.
S1.	Schematic of the Lab-built-based Raman setup	2
S2.	HRTEM-HAADF elemental analysis of the prepared nanoparticles	2
S3.	Photograph of the Scotch tape mask used for selective attachment of AuNPs	3
S4.	Droplet splitting of the saliva on PDAP at different inclination angles	3
S5.	Comparison of Raman spectra of dyes on Plain quartz as well as developed plasmonic droplet assay platform.	3
S6.	Raman Spectra of milk adulterants in pure form	4
S7.	Raman Spectra of different concentrations of Urea	4
S8	Raman Spectra of different concentrations of ammonium sulfate	5
S9.	Raman Spectra of different concentrations of melamine	5
ST1	Raman peak assignments of Rhodamine 6g	5
ST2	Raman peak assignments of Crystal Violet	6
ST3	Comparison of detection levels of Rh6G and CV	7
ST4	Raman peak assignments of Urea	7
ST5	Raman peak assignments of Ammonium sulfate	8
ST6	Raman peak assignments of Melamine	8
SV1	Movement of a 10 μl droplet on the oil-grafted quartz substrate at an inclination of 30°	-
SV2	Droplet splitting on PDAP at different inclination angles	-
SV3	Droplet splitting on PDAP with different surface tension liquids	-
	References	8

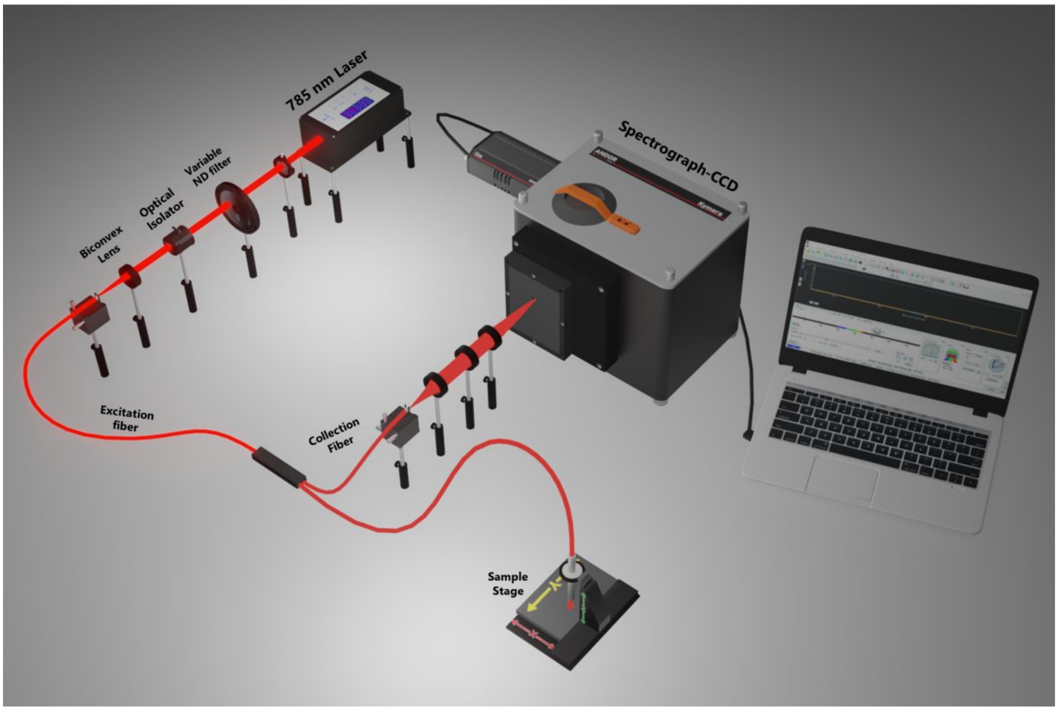

S1: Schematic of the Lab-built-based Raman setup

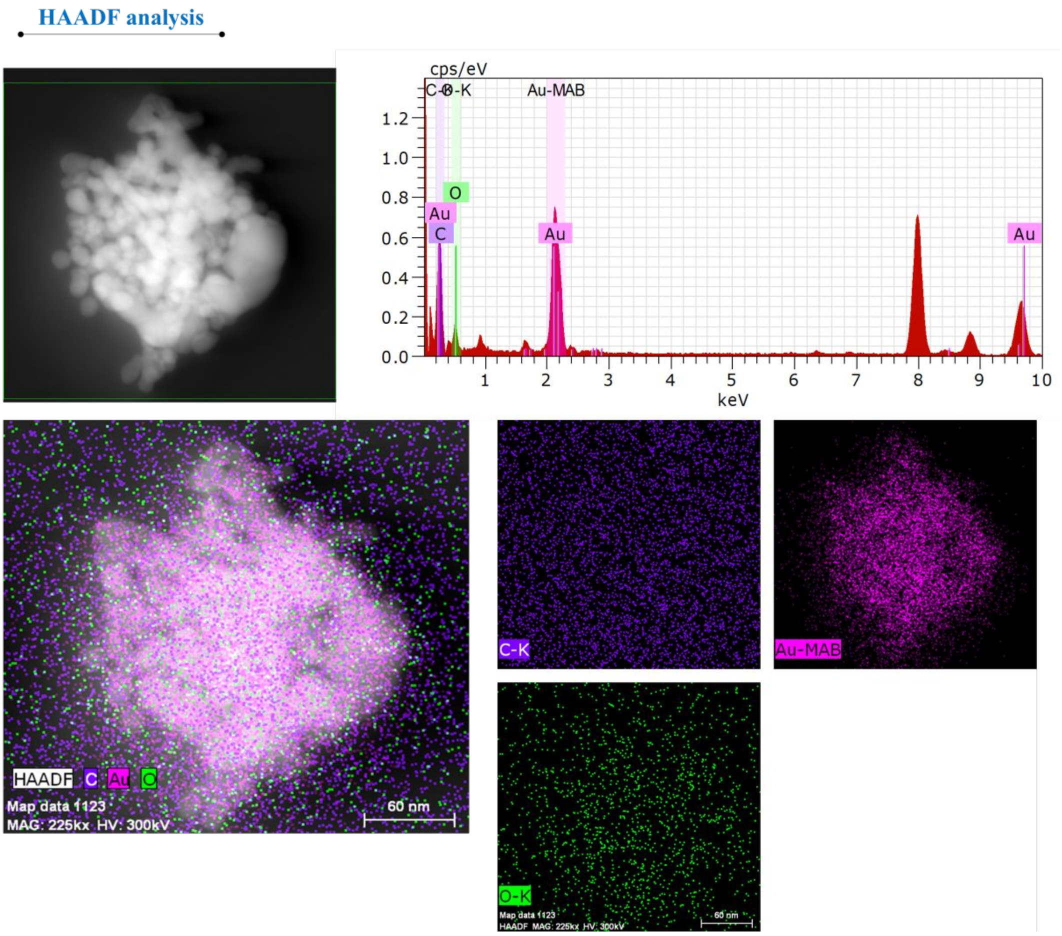

S2: HRTEM-HAADF analysis of the prepared nanoparticles showing the elemental composition of the prepared Au nanoparticles

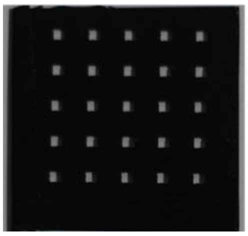

S3: Photograph of the Scotch tape mask with 1 X 1 mm rectangular regions used for selective attachment of AuNPs

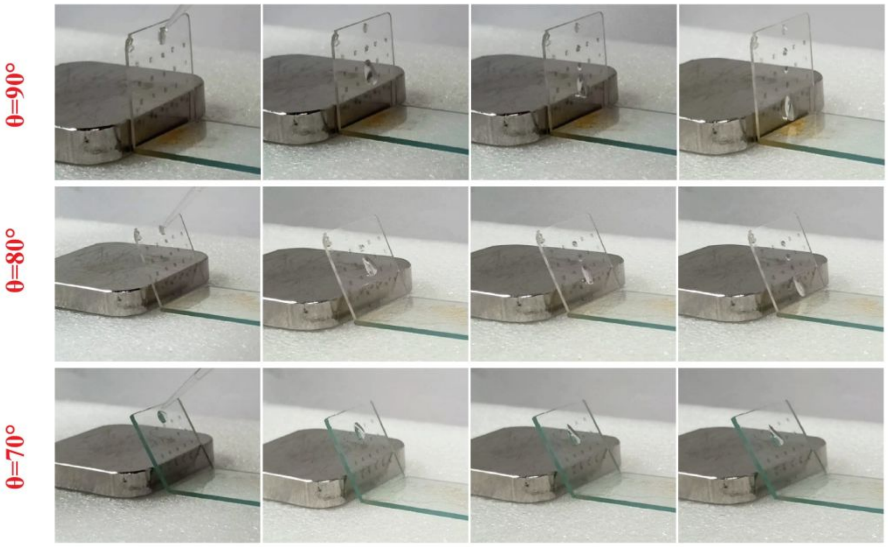

S4: Droplet splitting of the saliva on the developed PDAP placed at different inclination angles

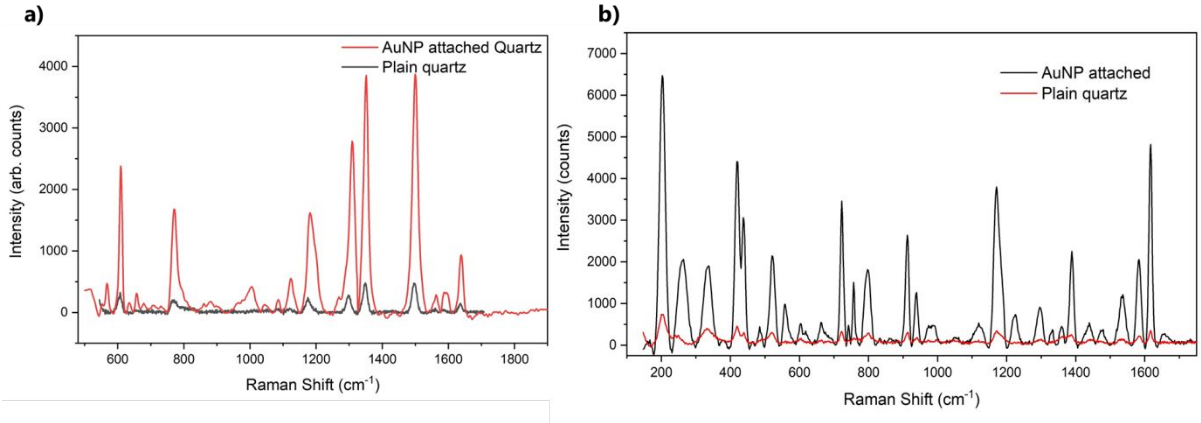

S5: Comparison of Raman spectra of a) Rh6G and b) CV on Plain quartz as well as developed plasmonic droplet assay platform.

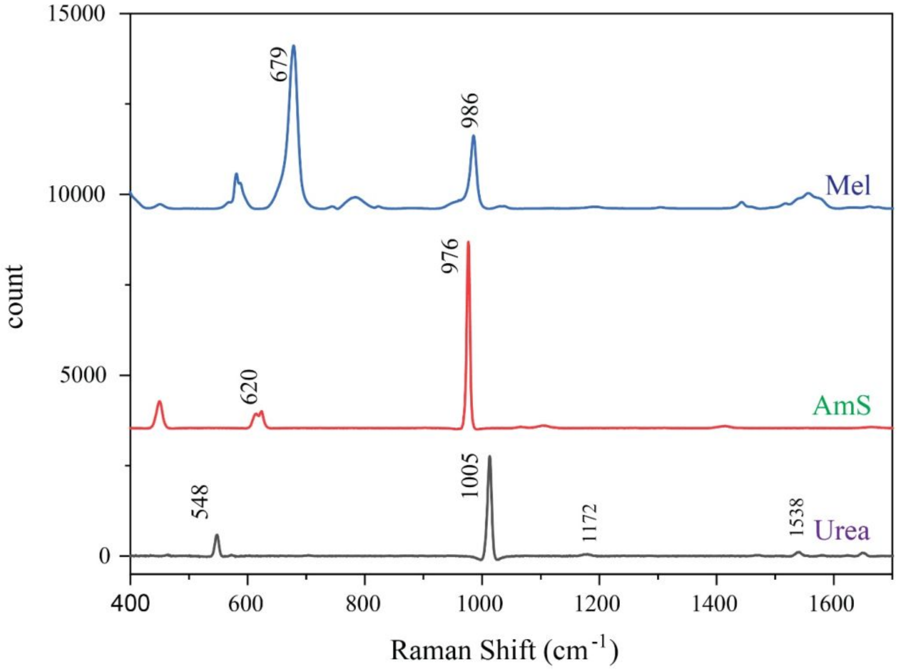

S6 a) Raman spectra of the milk adulterants used in the study urea, ammonium sulfate, and melamine in their pure form

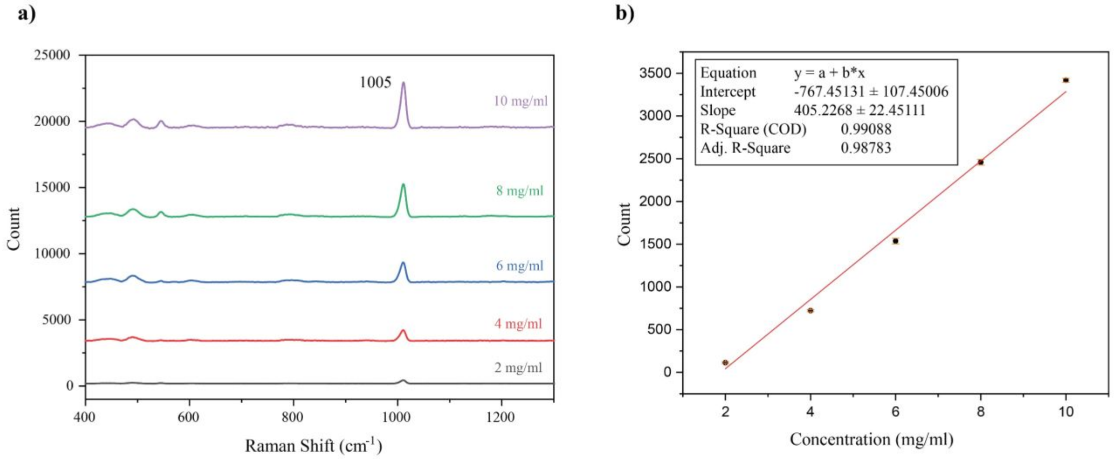

S7. a) Raman spectra of aqueous solution of urea of different concentrations b) linear plot showing the variation of the 1005 cm^{-1} Raman peak of urea

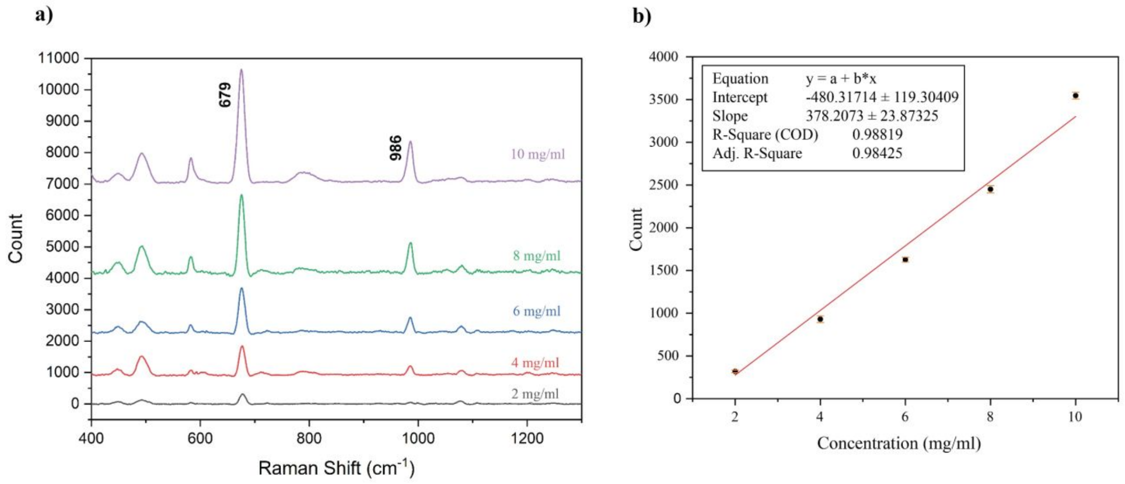

S8. a) Raman spectra of the aqueous solution of ammonium sulfate of different concentrations b) linear plot showing the variation of the 1005 cm^{-1} Raman peak of ammonium sulfate.

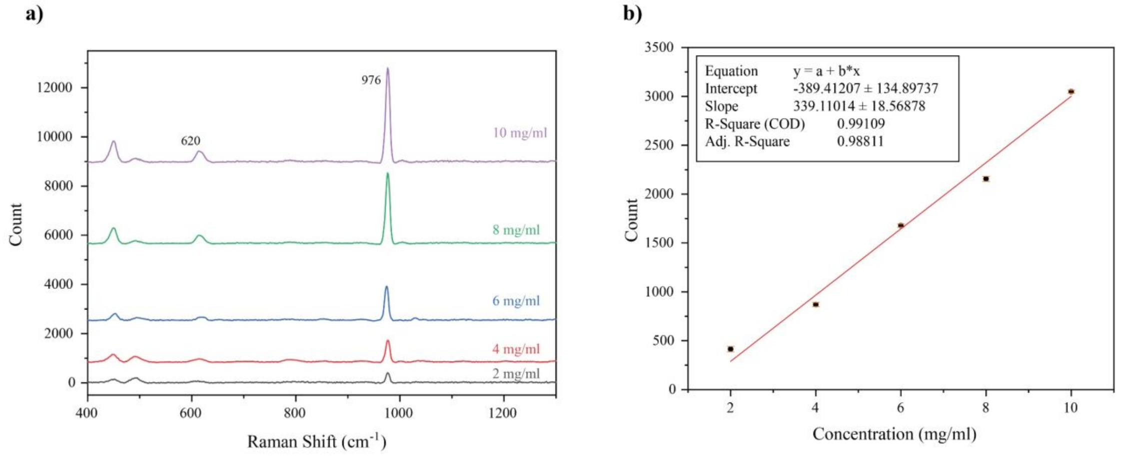

S9. a) Raman spectra of aqueous solution of melamine of different concentrations b) linear plot showing the variation of the 1005 cm^{-1} Raman peak of melamine.

ST1: Raman peak assignments of Rhodamine 6g

Raman Shift(cm^{-1})	Peak Assignment[1, 2]
612	C-C ring in-plane bending of xanthene/phenyl rings
775	C-H out of plane bending
1128	C-H in-plane bending of xanthene/phenyl rings
1182	C-H in-plane bending in xanthene ring
1309	Aromatic C-C streching
1358	C-C stretching in xanthene ring
1507	C-C stretching in xanthene ring
1577	C-C stretching in phenyl ring

1601	Hybrid mode (phenyl ring with $COOC_2H_5$)
1646	C-C stretching in xanthene ring

ST2: Raman peak assignments of Crystal Violet

Raman Shift(cm^{-1})	Assignments [2, 3]
339	Asymmetric bending of the phenyl C-phenyl, CH3 rocking, out of plane deformation of the C-N-C
413	Out–of–plane bending mode of C-C_{center}-C
434	Bending mode of C-N-C
524	Bending mode of C-N-C
568	Out-of-plane aromatic C-C deformation
725	C-N-C symmetric stretching vibration
750	Asymmetric 10a benzene mode
802	Ring C-H bending
905	C-H out-of-plane bending modes
1175	C-H in-plane bending mode
1391	Stretching vibration of nitrogen and phenyl ring
1530	Asymmetric stretching of phenyl ring and nitrogen
1584	In-plane aromatic C-C
1615	In-plane aromatic C-C

ST3: Comparison of detection level of Rhodamine 6G and crystal violet achieved on some of the reported different types of SERS substrates

Rhodamine 6G		Crystal Violet	
SERS substrate	***Detection Limit***	***SERS substrate***	***Detection Limit***
Thin films of rGO/AgNP hybrids	~1 nM [4]	Monolayer array of silica@Au core-shell nanoparticles	~10 nM [5]
Ag NPs arrayed on WO_{3-x} nanoflakes	8.5 pM [6]	Ag coated cellophane	~1nM [7]
TiO_2-Ag-GO composite	~10 nM [8]	TiO_2-Ag-GO composite	~1nM [8]
AgNPs/poly(3-caprolactone) nanofiber membrane	~1 pM [9]	AgNPs/poly(3-caprolactone) nanofiber membrane	~10 pM [9]
Porous molecularly imprinted polymer decorated with AuNP array	~0.1 nM [10]	Ag nanowires arrays	~1 fM [11]
Optically printed Ag nanoparticle on quartz	323 fM [2]	Optically printed Ag nanoparticle on quartz	82 pM [2]
Superhydrophobic gold-loaded nanoporous anodic alumina	146.3 pM [12]	AgNP decorated paper	~10 fM [13]
Gold nanoparticles decorated 2D-WSe_2	1 nM [14]	Hydrophobic slippery Teflon with AuNPs	1 pM [15]
Ag/ZnO/Ag hybrid	0.1 pM [16]	Au nanostars-based paper SERS substrate	1nM [17]
AuNP attached on quartz	~134 pM (This work)	AuNP attached on quartz	~10.1 nM (This work)

ST4: Raman peak assignments of Urea

**Raman Shift(cm^{-1})**	**Peak Assignment [18-20]**
548	CO deformation
1005	N-C-N stretching
1173	NH_2 symmetric vibrations
1468	NCN antisymmetric vibration
1538	CO symmetric stretch
1648	NH_2 symmetric deformation

ST5: Raman peak assignments of Ammonium sulfate

**Raman Shift(cm^{-1})**	**Peak Assignment [21,22]**
449	Symmetric bending of SO_4^{2-}
620	triply degenerate SO_4^{2-}deformation mode
976	symmetric stretch mode of SO_4^{2-}

ST6: Raman peak assignments of Melamine

**Raman Shift(cm^{-1})**	**Peak Assignment [23,24]**
679	in-plane deformation of the triazine ring
986	C–N stretching of the triazine ring.
1444	A_1 vibration of the triazine ring

SV1: Movement of a 10 µl droplet on the oil-grafted quartz substrate at an inclination of 30°

SV2: Droplet splitting on PDAP at different inclination angles

SV3: Droplet splitting on PDAP with different surface tension liquids

References:

(1) He, X. N.; Gao, Y.; Mahjouri-Samani, M.; Black, P. N.; Allen, J.; Mitchell, M.; Xiong, W.; Zhou, Y. S.; Jiang, L.; Lu, Y. F. Surface-enhanced Raman spectroscopy using gold-coated horizontally aligned carbon nanotubes. *Nanotechnology* **2012**, *23* (20), 205702.
(2) Monisha, K.; Suresh, K.; Bankapur, A.; George, S. D. Optical printing of plasmonic nanoparticles for SERS studies of analytes and thermophoretically trapped biological cell. *Sens Actuators, B Chem* **2023**, *377,* 133047.
(3) Mao, A.; Jin, X.; Gu, X.; Wei, X.; Yang, G. Rapid, green synthesis and surface-enhanced Raman scattering effect of single-crystal silver nanocubes. *J. Mol. Struct.* **2012**, *1021*, 158-161.
(4) Kavitha, C.; Bramhaiah, K.; John, N. S.; Ramachandran, B. E. Low cost, ultra-thin films of reduced graphene oxide–Ag nanoparticle hybrids as SERS based excellent dye sensors. *Chemical Physics Letters* **2015**, *629*, 81-86.
(5) Gu, C.; Man, S.-Q.; Tang, J.; Zhao, Z.; Liu, Z.; Zheng, Z. Preparation of a monolayer array of silica@gold core-shell nanoparticles as a SERS substrate. *Optik* **2020**, *221*, 165274.
(6) Gao, F.; Kong, W.; He, G.; Guo, Y.; Liu, H.; Zhang, S.; Yang, B. SERS-active vertically aligned silver/tungsten oxide nanoflakes for ultrasensitive and reliable detection of thiram. *Microchemical Journal* **2021**, *165*, 106046.
(7) Wei, W.; Huang, Q. Preparation of cellophane-based substrate and its SERS performance on the detection of CV and acetamiprid. *Spectrochimica Acta Part A: Molecular and Biomolecular Spectroscopy* **2018**, *193*, 8-13.
(8) Zhang, M.; Sun, H.; Chen, X.; Zhou, H.; Xiong, L.; Chen, W.; Chen, Z.; Bao, Z.; Wu, Y. The influences of graphene oxide (GO) and plasmonic Ag nanoparticles modification on the SERS sensing performance of TiO2 nanosheet arrays. *Journal of Alloys and Compounds* **2021**, *864*, 158189.
(9) Shi, J.; You, T.; Gao, Y.; Liang, X.; Li, C.; Yin, P. Large-scale preparation of flexible and reusable surface-enhanced Raman scattering platform based on electrospinning AgNPs/PCL nanofiber membrane. *RSC Advances* **2017**, *7* (75), 47373-47379.

(10) Wang, J.; Li, J.; Zeng, C.; Qu, Q.; Wang, M.; Qi, W.; Su, R.; He, Z. Sandwich-Like Sensor for the Highly Specific and Reproducible Detection of Rhodamine 6G on a Surface-Enhanced Raman Scattering Platform. *ACS Applied Materials & Interfaces* **2020**, *12* (4), 4699-4706.
(11) Wang, K.; Qiu, Z.; Qin, Y.; Feng, L.; Huang, L.; Xiao, G. Preparation and SERS performance of silver nanowires arrays on paper by automatic writing method. *Spectrochimica Acta Part A: Molecular and Biomolecular Spectroscopy* **2022**, *281*, 121580.
(12) Choudhari, K. S.; Sinha, R. K.; Kulkarni, S. D.; Santhosh, C.; George, S. D. Facile fabrication of superhydrophobic gold loaded nanoporous anodic alumina as surface-enhanced Raman spectroscopy substrates. *Journal of Optics* **2022**, *24* (4), 044002.
(13) Verma, M.; Naqvi, T. K.; Tripathi, S. K.; Kulkarni, M. M.; Dwivedi, P. K. Paper based low-cost flexible SERS sensor for food adulterant detection. *Environmental Technology & Innovation* **2021**, *24*, 102033.
(14) Majumdar, D.; Jana, S.; Kumar Ray, S. Gold nanoparticles decorated 2D-WSe2 as a SERS substrate. *Spectrochimica Acta Part A: Molecular and Biomolecular Spectroscopy* **2022**, *278*, 121349.
(15) Zhang, D.; You, H.; Yuan, L.; Hao, R.; Li, T.; Fang, J. Hydrophobic Slippery Surface-Based Surface-Enhanced Raman Spectroscopy Platform for Ultrasensitive Detection in Food Safety Applications. *Analytical Chemistry* **2019**, *91* (7), 4687-4695.
(16) Pal, A. K.; Chandra, G. K.; Umapathy, S.; Bharathi Mohan, D. Ultra-sensitive, reusable, and superhydrophobic Ag/ZnO/Ag 3D hybrid surface enhanced Raman scattering substrate for hemoglobin detection. *Journal of Applied Physics* **2020**, *127* (16), 164501.
(17) He, S.; Chua, J.; Tan, E. K. M.; Kah, J. C. Y. Optimizing the SERS enhancement of a facile gold nanostar immobilized paper-based SERS substrate. *RSC Advances* **2017**, *7* (27), 16264-16272.
(18) Keuleers, R.; Desseyn, H.; Rousseau, B.; Van Alsenoy, C. J. T. J. o. P. C. A. Vibrational analysis of urea. **1999**, *103* (24), 4621-4630.
(19) Khan, K. M.; Krishna, H.; Majumder, S. K.; Gupta, P. K. Detection of Urea Adulteration in Milk Using Near-Infrared Raman Spectroscopy. *Food Anal. Methods.* **2015**, *8* (1), 93-102.
(20) Ren, M.; Arnold, M. A. Comparison of multivariate calibration models for glucose, urea, and lactate from near-infrared and Raman spectra. *Anal. Bioanal. Chem.* **2007**, *387* (3), 879-888.
(21) Nieuwoudt, M. K.; Holroyd, S. E.; McGoverin, C. M.; Simpson, M. C.; Williams, D. E. Raman spectroscopy as an effective screening method for detecting adulteration of milk with small nitrogen-rich molecules and sucrose. *J. Dairy Sci.* **2016**, *99* (4), 2520-2536.
(22) Qiu, J.; Li, X.; Qi, X. Raman Spectroscopic Investigation of Sulfates Using Mosaic Grating Spatial Heterodyne Raman Spectrometer. *IEEE Photonics Journal* **2019**, *11* (5), 1-12.
(23) Koglin, E.; Kip, B. J.; Meier, R. J. Adsorption and displacement of melamine at the Ag/electrolyte interface probed by surface-enhanced Raman microprobe spectroscopy. *Journal of Physical Chemistry* **1996**, *100* (12), 5078-5089.
(24) Liu, X.; Zinin, P.; Ming, L.; Acosta, T.; Sharma, S.; Misra, A.; Hong, S. Raman spectroscopy of melamine at high pressures. In *Journal of Physics: Conference Series*, 2010; IOP Publishing: Vol. 215, p 012045.
